# Granulated Wheat Biscuit

**Published:** 1875-11

**Authors:** 


### GRANULATED WHEAT BISCUIT

The sale of this excellent food is increasing very rapidly, and the store of the Health Food Company, 137 East Eighth Street, is thronged with callers who desire to examine the product and inquire as to the methods employed in its manufacture. As seeing is believing, all who investigate, buy; and thus the trade shows great activity. We are glad to note this, because we thoroughly approve of the pure and nutritious foods prepared by this company, and feel that we do a great service to the world in urging their use upon all. If people would live on such foods, the race of man would be greatly improved.

---

MAKE no more vows to perform this or that. It shows no great strength, and makes thee ride behind thyself.

